# Improvements to and Comparison of Static Terrestrial LiDAR Self-Calibration Methods

**DOI:** 10.3390/s130607224

**Published:** 2013-05-31

**Authors:** Jacky C. K. Chow, Derek D. Lichti, Craig Glennie, Preston Hartzell

**Affiliations:** 1 Department of Geomatics Engineering, the University of Calgary, 2500 University Drive N.W., Calgary, AB T2N 1N4, Canada; E-Mail: ddlichti@ucalgary.ca; 2 Department of Civil and Environmental Engineering, the University of Houston, N107 Engineering Building 1, Houston, TX 77204-4003, USA; E-Mails: clglennie@uh.edu (C.G.); pjhartzell@uh.edu (P.H.)

**Keywords:** LiDAR, terrestrial laser scanners, calibration, accuracy, error analysis, quality assurance

## Abstract

Terrestrial laser scanners are sophisticated instruments that operate much like high-speed total stations. It has previously been shown that unmodelled systematic errors can exist in modern terrestrial laser scanners that deteriorate their geometric measurement precision and accuracy. Typically, signalised targets are used in point-based self-calibrations to identify and model the systematic errors. Although this method has proven its effectiveness, a large quantity of signalised targets is required and is therefore labour-intensive and limits its practicality. In recent years, feature-based self-calibration of aerial, mobile terrestrial, and static terrestrial laser scanning systems has been demonstrated. In this paper, the commonalities and differences between point-based and plane-based self-calibration (in terms of model identification and parameter correlation) are explored. The results of this research indicate that much of the knowledge from point-based self-calibration can be directly transferred to plane-based calibration and that the two calibration approaches are nearly equivalent. New network configurations, such as the inclusion of tilted scans, were also studied and prove to be an effective means for strengthening the self-calibration solution, and improved recoverability of the horizontal collimation axis error for hybrid scanners, which has always posed a challenge in the past.

## 1. Introduction

Three-dimensional point clouds from terrestrial laser scanning (TLS) instruments are a valuable asset for a variety of real-world problems. Beside their traditional use in areas such as digital terrain modelling, geological exploration, surveying, archeological and architectural documentation, they have found applications in forensics and crime scene investigations, deformation monitoring of dams, buildings and landslides, and more recently for scanning movie sets. For many applications of TLS, it is assumed that the point cloud is error-free. Although this is far from reality, numerous efforts have attempted to produce more reliable and accurate point clouds. New and improved filters and measurement algorithms have limited a great deal of outliers and noise in the point cloud. Typical TLS projects involve grouping common points to model a surface or object to reduce the effect of random noise. Modern TLS instruments behave much like total stations; however their systematic errors cannot be eliminated by taking direct and reverse measurements. Instead, users usually have to rely on the manufacturer's black box instrument calibration. The manufacturer's calibration has been shown to be an effective method for improving the scanner's observation precision, but it has also been demonstrated that point-based user self-calibration can further reduce the standard deviation of the scanners' observations [1].

Some noticeable advantages of the TLS point-based self-calibration method include:

- Manufacturer-independent total system error modelling.
- Frequent and rapid calibration at the user's convenience for quality assurance.
- A common basis for comparing the measurement precision of different scanners.

In the past, point-based self-calibration has proven to be successful at identifying and modelling systematic errors in a variety of TLS instruments [2-5]. It can also be executed efficiently once the point clouds are captured; for example the point targets can be extracted and matched automatically (e.g., Leica Cyclone) and the selection of the relevant error model parameters can be made with minimal user interaction [6]. However, its largest drawbacks are the time and cost required to set up a large quantity of signalised targets, and the poor recovery of the horizontal collimation axis error in hybrid-type scanners. Self-printed paper targets could substitute for the expensive laser scanner targets [7], but it might still be a challenge to install targets in large rooms [8] despite the fact that they are beneficial to the calibration [9].

An alternative is to replace signalised targets with well-defined geometric features that are commonly encountered by the user. In [10] the authors presented mathematical models for simultaneous registration and modelling of objects that are frequently encountered at industrial sites. These mathematically well-defined surfaces include planes, cylinders, tori, and spheres. This paper concentrates on the utilization of planes for performing TLS self-calibration. The main motivations of this paper are: (1) to verify whether or not point-based and plane-based TLS self-calibration produce equivalent results; and (2) to propose new network configuration measures (e.g., the inclusion of tilted scans) to enhance the quality of TLS calibration. Special attention is placed on the precise estimation of the collimation axis error for hybrid-type scanners that has proven difficult to resolve in the past [3,8,9,11].

This article is organized as follows: in Section 2, previous research on TLS error modelling is reviewed. The functional and stochastic models used for carrying out the point-based and plane-based error modelling are explained in Section 3. Section 4 shows simulated calibrations using the point-based and plane-based methods. Cases where only levelled scans are used and when levelled and tilted scans are used in conjunction are analyzed in terms of the standard deviation and correlation of the systematic errors. Finally, Section 5 shows point-based and plane-based calibration results from TLS instruments built by Riegl, Z+F, Leica, and Trimble.

## 2. Previous Work

Unmodelled systematic distortions have been identified in TLS instruments from different manufacturers having unique internal components and architectures [2,12–15]. Previous calibration attempts have modelled the scanner based on the panoramic camera model [16], theodolites [17] and total stations [18,19]. The two former approaches are restricted to modelling systematic errors in the angular measurements and only the last method can simultaneously account for systematic errors in distances and directions. This desirable trait makes the third option the preferred approach.

Distance measurements in survey grade TLS instruments are achieved by measuring the two-way travel time of laser pulse(s) or the phase difference of amplitude modulated continuous light waves. Many of the errors in the Electronic Distance Measurement Instruments (EDMI) are well known and have been shown to be applicable to laser scanners [20]. To determine the 3D position of a single point, the laser needs to be redirected in two orthogonal directions. This can be achieved by deflecting the laser beam using a mirror device (e.g., oscillating, rotating, or polygonal mirror), physically rotating the scanner head, or using multiple rangefinders with fixed angular separations. The methodology used for sweeping the laser over the scene has a strong influence on the resulting field of view (FOV) of the TLS instrument. For example, scanners with oscillating mirrors (e.g., Trimble GX and Leica Scanstation2) usually have a much smaller vertical FOV than scanners using rotating mirrors (e.g., FARO Focus^3D^, Z+F Imager 5010, Leica P20, and Trimble TX8).

According to [21], TLS instruments can be broadly categorized into hybrid-type and panoramic-type based on their FOV. The calibration of panoramic-type scanners is better understood because it allows data to be acquired in two layers (*i.e.*, in front of and behind the unit). This is comparable to total stations observing in both the face-left and face-right orientations; the end result is parameter de-correlation and better model identification. In [18] the systematic errors in a panoramic-type scanner, Faro 880, were studied in great detail and an extensive 17 parameter error model with both physical and empirical terms that is independent of the scanner architecture was presented. This method solves for the exterior orientation parameters (EOPs), object space target coordinates, and additional parameters (APs) simultaneously in a free station network adjustment, rather than solving for the systematic errors one at a time [17].

Further analyses in [8] identified and explored the correlation of APs with other parameters in self-calibration. It has been shown that problems with inflated correlation between parameters are largely mitigated by having independent measurements of the EOPs (e.g., levelling the scanner using inclinometer measurements). Levelling information is valuable—even if its quality is low—for the recovery of the vertical circle index error. Parameter de-correlation is most important for calibrating hybrid-type scanners, which suffer from stronger dependencies than panoramic scanners due to their data acquisition pattern (*i.e.*, data can only be captured in one face). This has been independently confirmed by [19] who studied the self-calibration of many different hybrid-type TLS systems. Instead of defining the datum using inner constraints, which is known to have the negative effect of giving higher parameter correlation [22], all parameters are treated as observations in a unified least-squares adjustment in [19]. Although the inclusion of independent observations of the EOPs can help de-correlate many of the parameters, it also can magnify the complexity of the calibration procedure and diminishes its ease of use. For example, to recover the rangefinder offset accurately, the position of the scanner needs to be measured with accuracy better than one millimetre. In practice, this is difficult to achieve unless stable pillars with known coordinates are used. Nonetheless, [9,19] have clearly explained the limitations of the current methodologies to solve for the horizontal collimation axis error, which is perfectly correlated with the tertiary rotation angle for hybrid-type scanners. High parameter correlation can pose a threat to data integrity if the scanner is not calibrated *in-situ* [23]. In [11], the correlation between the collimation axis error and the heading angle of the scanner was reduced through the introduction of a new mathematical model for the AP as well as approximating the relative heading angle between scans. The correlation was successfully reduced but the standard deviation of the recovered systematic error was still higher than expected (*i.e.*, in the order of arc minutes). To date, a solution to this issue has not yet been tested thoroughly and reported.

Another approach to laser scanner calibration focuses on minimizing the distance between measured points and a well-defined mathematical surface. In general, any geometric features that can be used for registration can be extended for self-calibration [10]. However, most calibration routines have focused on the use of planes, likely because of their simple mathematical representation and abundance in urban settings. This concept was first presented by [24] for calibrating their in-house laser scanner. In [25] it was studied further as an extension of their point-based calibration model for panoramic-type scanners. Through simulation they investigated different scanner configurations for performing plane-based calibration and suggested that a long baseline is helpful for recovering the collimation axis error when only two scans are acquired. [26] showed through simulation that the impact of the four fundamental systematic errors (*i.e.*, rangefinder offset, vertical circle index error, trunnion axis error, and horizontal collimation axis error) on the measurement residuals is very similar between the point-based and plane-based calibration. They also indicated some subtle differences between the two methods; in particular, the correlation between APs and EOPs needs to be further studied.

The Velodyne HDL-64E S2 scanner was calibrated using planar-features and this improved the geometric quality of the point cloud by a factor of three [27]. The inclusion of tilted scans in the adjustment was tested in [27,28] for reducing the correlation between horizontal offset and horizontal angular offset as well as vertical offset and vertical angular offset in the Velodyne calibration. [11] also tried tilting the Trimble GS200 by a small amount (<10°) and reported minor improvements in recovery of the horizontal collimation axis error. The use of planar features for calibrating laser scanners can also be found in airborne laser scanning. The authors of [29] presented the plane-based calibration results of a mobile scanning system mounted on a helicopter and have indicated the benefits of having tilted planes with various orientations for recovering the boresight angles and rangefinder offset. In [30] planes were used to solve for the internal calibration parameters and boresight angles of a mobile terrestrial scanner (Velodyne). Other geometric features such as cylinders and catenaries have also been used for calibrating static terrestrial laser scanners [31] and mobile terrestrial laser scanners [32], respectively.

## 3. Mathematical Model

The 3D Cartesian coordinate of every point in a TLS point cloud is determined from a distance observation and two angular observations. The conversion between the spherical coordinate system, in which the observations are made, and the Cartesian coordinate system, in which the point cloud is typically defined, is given by Equation (1). Every point is uniquely determined in TLS, and therefore the same point needs to be observed more than once to achieve any redundancy. Having redundancy in the solution is crucial for quality assurance purposes when registering scans and calibrating the scanner. This is rather difficult to achieve in TLS because the points are irregularly spaced and in general there are no point-to-point correspondences between scans. The conventional approach is to measure multiple points on the surface of a signalised target and calculate the centre of the target. Corresponding target centroids are used to relate scans captured from different positions and orientations, usually via a 3D rigid body transformation (Equation (2)). For the point-based registration model, one can substitute Equation (1) into Equation (2) and estimate the EOPs, which ensure best fit between the targets in object space. In other words, the 3D volume of uncertainty at every object space target position is minimized.

Alternatively, geometric features can be used to solve the correspondence problem. Features such as planes, spheres, tori, cylinders, pyramids, and catenaries can be used for registration. If planes are used, the orthogonal distance between every point observed on the plane is minimized (Equation (3)). The datum in both point-based and plane-based self-calibrations is defined by enforcing inner constraints on the object space primitives. For the plane-based calibration, the imposition of inner constraints on the plane parameters has been shown to be the preferred datum definition because it mitigates correlation problems [33]. In registration, if a highly redundant strong network of well-distributed targets is used, APs can be appended to the observations to model systematic errors in the scanner. APs can be categorised into physical terms (the source of error is known) and empirical terms (the source of error is unknown). The known physical APs in the range, horizontal direction, and vertical angle measurements are given by Equations 4–6 respectively. In both point-based and plane-based self-calibration, the sum of the weighted squared residuals is minimized while simultaneously solving for the EOPs, APs, and object space target coordinates or feature parameters. Details about the least squares estimation method can be found in [34]:

(1) $$\begin{array}{l} \rho_{ij}=\sqrt{x_{ij}^{2}+y_{ij}^{2}+z_{ij}^{2}}+\Delta \rho \\ \theta_{ij}=\tan^{-1}\;(\frac{y_{ij}}{x_{ij}}\;)+\Delta \theta \\ \alpha_{ij}=\tan^{-1}\;(\frac{z_{ij}}{\sqrt{x_{ij}^{2}+y_{ij}^{2}}}\;)+\Delta \alpha \end{array}$$

where

*ρ_ij_*, *θ_ij_* and *α_ij_* are the slope distance, horizontal direction, and vertical angle of point i in scanner space j
*x_ij_, y_ij_*, and *z_ij_* are the Cartesian coordinates of point i in scanner space j
Δ*ρ*, Δ*θ*, and Δα are the additional parameters for the scanner

(2) $$\left[\begin{array}{c} x_{ij}\\ y_{ij}\\ z_{ij} \end{array}\right]=R_{3}\;(k_{j}\;)R_{2}\;(\phi_{j}\;)R_{1}\;(\omega_{j}\;)\;(\left[\begin{array}{c} X_{i}\\ Y_{i}\\ Z_{i} \end{array}\right]-\left[\begin{array}{c} X_{oj}\\ Y_{oj}\\ Z_{oj} \end{array}\right]\;)$$

where

*X_i_, Y_i_*, and *Z_i_* are the Cartesian coordinates of point i in object space
*X_oi_, Y_oi_*, and *Z_oi_* are the origin of scanner space j in object space
*ω_j_*, *ϕ_j_*, and *k_j_* are the orientation of scanner space j relative to object space

(3) $$\;(a_{k}\;b_{k}\;c_{k}\;)\left\{R_{3}\;(k_{j}\;)R_{2}\;(\phi_{j}\;)R_{1}\;(\omega_{j}\;)\left[\begin{array}{c} \;(\rho_{ij}-\Delta \rho \;)\cos\;(\alpha_{ij}-\Delta \alpha \;)\cos\;(\theta_{ij}-\Delta \theta \;)\\ \;(\rho_{ij}-\Delta \rho \;)\cos\;(\alpha_{ij}-\Delta \alpha \;)\sin\;(\theta_{ij}-\Delta \theta \;)\\ \;(\rho_{ij}-\Delta \rho \;)\sin\;(\alpha_{ij}-\Delta \alpha \;) \end{array}\right]+\left[\begin{array}{c} X_{oj}\\ Y_{oj}\\ Z_{oj} \end{array}\right]\right\}-d_{k}=0$$

where *a_k_, b_k_, c_k_*, and *d_k_* are the plane parameters defining the normal axis and orthogonal distance to the plane

(4) $$\Delta \rho =A_{0}+A_{1}\rho_{ij}+A_{2}\sin\;(\alpha_{ij}\;)+A_{3}\sin\;(\frac{4\pi }{U}\rho_{ij}\;)+A_{4}\cos\;(\frac{4\pi }{U}\rho_{ij}\;)+ET_{\rho }$$

where

*A_0_* is the rangefinder offset
*A_1_* is the range scale factor error
*A_2_* is the laser axis vertical offset
*A_3_* and A_4_ are the cyclic errors
*U* is the unit length
*ET_ρ_* is the empirical range errors

(5) $$\begin{array}{c} \Delta \theta =B_{1}\theta +B_{2}\sin\;(\theta \;)+B_{3}\cos\;(\theta \;)+B_{4}\sin\;(2\theta \;)+B_{5}\cos\;(2\theta \;)+B_{6}\sec\;(\alpha \;)*\\ +B_{7}\tan\;(\alpha \;)+B_{8}\rho^{-1}+B_{9}\sin\;(\alpha \;)+B_{10}\cos\;(\alpha \;)+ET_{\theta }\\ {}*\text{Note: for hybrid-type scanners, the reduced collimation model is used instead},B_{6}\;[\sec(\alpha )-1] \end{array}$$

where

*B_1_* is the horizontal direction scale factor error
*B_2_* and *B_3_* are the horizontal circle eccentricity
*B_4_* and *B_5_* are the non-orthogonality of horizontal encoder and vertical axis
*B_6_* is the horizontal collimation axis error
*B_7_* is the trunnion axis error
*B_8_* is the horizontal eccentricity of collimation axis
*B_9_* and *B_10_* are the trunnion axis wobble
*ET_θ_* is the empirical horizontal direction errors

(6) $$\Delta \alpha =C_{0}+C_{1}\alpha +C_{2}\sin\;(\alpha \;)+C_{3}\cos\;(\alpha \;)+C_{4}\sin\;(2\alpha \;)+C_{5}\cos\;(2\alpha \;)+C_{6}\rho^{-1}+C_{7}\sin\;(\theta \;)+C_{8}\cos\;(\theta \;)+ET_{\alpha }$$

where

*C_0_* is the vertical circle index error
*C_1_* is the scale factor error
*C_2_* and *C_3_* are the vertical circle eccentricity
*C_4_* and *C_5_* are the non-orthogonality of vertical encoder and trunnion axis
*C_6_* is the vertical eccentricity of collimation axis
*C_7_* and *C_8_* is the vertical axis wobble
*ET_α_* is the empirical elevation angle errors

As documented in [8,9,11,19], weighted constraints can be added to the self-calibration to decouple some of the parameters, namely high correlations between the APs and EOPs. They can be easily integrated as linear pseudo-observation equations as shown in Equation (7). For the stochastic model, the three observations are assumed to be independent of each other. Correlations between observations can exist, especially if the observations are within close proximity of each other, which may be the case with newer scanners acquiring even denser point clouds than before. But this assumption of independent observations can simplify the adjustment model and allow the use of mathematical techniques such as the summation of normals [29]. Moreover, for point-based self-calibration this is largely mitigated by the spatial separation of the placement of signalised targets. For plane-based calibration this is more of a concern, but can also be reduced by downsampling the point cloud. The adopted stochastic model for the range and angular observations in this paper is shown in Equation (8). Both angular observations' standard deviations are assumed to be constant, while the range observation's standard deviation changes as a function of the incidence angle. This dependency is mainly due to the enlargement of the laser beam footprint at high incidence angle. Therefore a lower weight is assigned to range observations captured from an oblique angle [35]:

(7) $$\begin{array}{cc} \omega_{j}= & \omega_{\text{obs}}\pm \sigma_{\omega }\\ \phi_{j}= & \phi_{\text{obs}}\pm \sigma_{\phi }\\ k_{j}= & k_{\text{obs}}\pm \sigma_{k} \end{array}$$

(8) $$\begin{array}{c} E\left\{{ɛ}_{\rho }^{2}\right\}=\sigma_{\rho }^{2}\sec^{2}\;(\beta \;)\\ \text{E}\left\{{ɛ}_{\theta }^{2}\right\}=\sigma_{\theta }^{2}\\ \text{E}\left\{{ɛ}_{\alpha }^{2}\right\}=\sigma_{\alpha }^{2}\\ \text{E}\left\{{ɛ}_{\theta \alpha }^{2}\right\}=\text{E}\left\{{ɛ}_{\rho \alpha }^{2}\right\}=E\left\{{ɛ}_{\rho \theta }^{2}\right\}=0 \end{array}$$

where *β* is the incidence angle of the laser on the surface being measured.

Variance component estimation (VCE) has been applied to better characterize the relative weights between the three observation groups (*i.e., ρ, θ*, *α*). To reduce the chance of blunders, Baarda's data snooping is performed after the adjustment to identify outliers, which are subsequently removed. These tools have been widely adopted in conventional survey network adjustments and often adopted in point-based TLS self-calibration as well. However, for the plane-based self-calibration only Baarda's data snooping was implemented. It was performed first in scanner space during plane extraction and then again during the plane-based self-calibration. VCE was not used in this case because, as shown in Section 3, many observations have a zero-valued (or near zero) residuals, especially the angular observations. This is a drawback of using planes, because only observations made in the direction orthogonal to the planes are constrained in the adjustment. When considering a small plane whose normal is directed towards the origin of the scanner space, both angular observations are more or less perpendicular to the normal vector of the plane and do not contribute to the estimation of the plane parameters. This drawback can be found in other geometric features too, such as observations along the principal direction of a cylinder. These large quantities of zero residuals can bias the estimated variance components, resulting in optimistic standard deviation estimates for the observations. Therefore, in this paper the observation weights estimated from the point-based self-calibration were used and held fixed in the plane-based self-calibration.

## 4. Simulated Data: Results and Discussion

In this section, the propagation of systematic errors into the residuals of the observations for scanners having both panoramic and hybrid type architectures will be studied. This is rather important because the presented self-calibration method models the systematic error in the scanner's observation space. In [18], the model identification problem was mainly addressed by studying the residual plots of the observations and visualising the systematic trends. However, it has been reported that some systematic errors which cannot be visually identified in the observation residuals can still be modelled correctly; an example is the vertical circle index error and the trunnion axis error in hybrid type scanners [11]. In general, if a systematic error can be visually identified in the observation residuals, it can be recovered with greater confidence.

**Figure 1.**
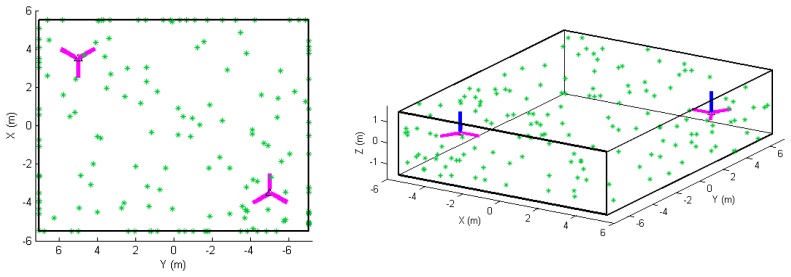
Network configuration of the simulated room: (left) top view and (right) oblique view. The blue line indicates the scanner's z-axis and magenta line indicates the scanner's x-axis (note that three scans were simulated at each of the two positions).

For this study, the APs presented in Equations 4–6 are tested one at a time in point-based and plane-based self-calibration for hybrid and panoramic scanners, with some exceptional sinusoidal terms being tested in pairs. A 14 m by 11 m by 3 m rectangular room with 120 randomly distributed signalized targets was simulated. This was chosen as a representation of common realistic calibration setups found in literature [11,18]. It has been shown that larger rooms which cover the scanner's minimum and maximum unambiguous range are ideal, but difficult to come across, especially when modern pulse-based TLS instruments can measure up to 6 km (e.g., the Riegl VZ-6000). Since it is known that systematic errors can be recovered better when both the horizontal and vertical angular field of view is maximized, no FOV restriction has been placed on the simulated scan data. This is justifiable as technological developments have continuously increased the FOV of scanners in the past decade — for example, the vertical FOV of the Trimble scanner increased from 60° to 320° (GX to TX8). The geometric arrangement of the targets/planes and scanner setups are shown in Figure 1. Six scans were simulated at two unique positions in the room. At both positions there were three leveled scans offset by 60° about the tertiary axis. A total of 120 points visible from every position were evenly distributed on each of the six planes. For the plane-based calibration, 20 points on each plane is a very low point density, but is done to ensure the same observations as point-based calibration are used. The distance and angular measurement noise was assumed to be 0.5 mm (at 0° incidence) and 20”, respectively. In the case where tilted scans were tested, two of the levelled scans from one of the corners were set to be tilted by −45° and +45°. Since the focus of this simulation is on model identification, the minimum number of scans/targets required for calibration is not addressed in this paper. All simulated range APs are 10 mm in magnitude with the range scale factor error being exaggerated to 2. The angular systematic errors are set to a magnitude of 3′, except for *B_8_* and *C_6_* which were 10 mm.

**Figure 2.**
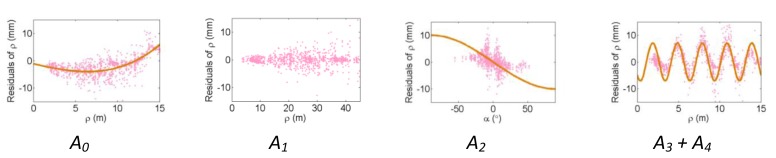
Residual plots of systematic errors in the range observations (*ρ*) from point-based self-calibration for hybrid-type scanners: *A_0_* is the rangefinder offset, *A_1_* is the range scale factor error, *A_2_* is the laser axis vertical offset, and *A_3_* + *A_4_* are the cyclic errors.

**Figure 3.**
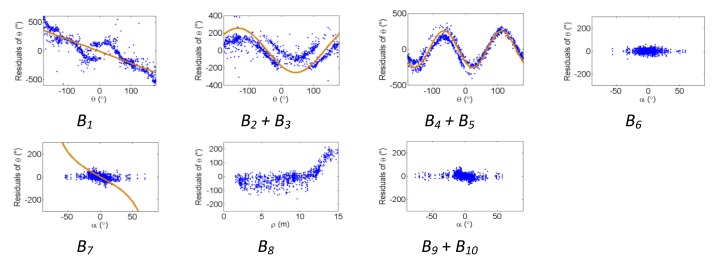
Residual plots of systematic errors in the horizontal angle observations (*θ*) from point-based self-calibration for hybrid-type scanners: *B_1_* is the horizontal direction scale factor error, *B_2_* + *B_3_* are the horizontal circle eccentricity, *B_4_* + *B_5_* are the non-orthogonality of horizontal encoder and vertical axis, *B_6_* is the horizontal collimation axis error, *B_7_* is the trunnion axis error, *B_8_* is the horizontal eccentricity of collimation axis, and *B_9_* + *B_10_* are the trunnion axis wobble.

**Figure 4.**
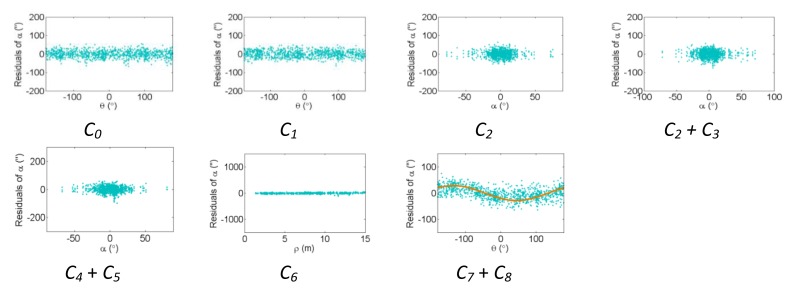
Residual plots of systematic errors in the vertical angle observations (*α*) from point-based self-calibration for hybrid-type scanners: *C_0_* is the vertical circle index error, *C_1_* is the scale factor error, *C_2_* + *C_3_* are the vertical circle eccentricity, *C_4_* + *C_5_* are the non-orthogonality of vertical encoder and trunnion axis, *C_6_* is the vertical eccentricity of collimation axis, and *C_7_* + *C_8_* is the vertical axis wobble.

**Figure 5.**
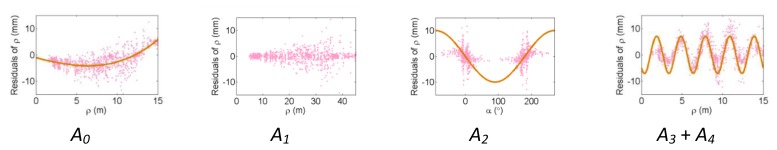
Residual plots of systematic errors in the range observations (*ρ*) from point-based self-calibration for panoramic-type scanners: *A_0_* is the rangefinder offset, *A_1_* is the range scale factor error, *A_2_* is the laser axis vertical offset, and *A_3_* + *A_4_* are the cyclic errors.

**Figure 6.**
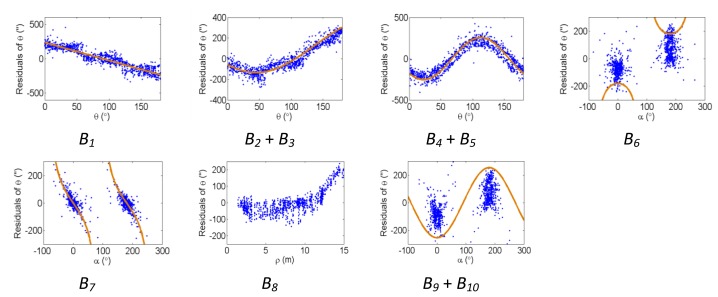
Residual plots of systematic errors in the horizontal angle observations (*θ*) from point-based self-calibration for panoramic-type scanners: *B_1_* is the horizontal direction scale factor error, *B_2_* + *B_3_* are the horizontal circle eccentricity, *B_4_* + *B_5_* are the non-orthogonality of horizontal encoder and vertical axis, *B_6_* is the horizontal collimation axis error, *B_7_* is the trunnion axis error, *B_8_* is the horizontal eccentricity of collimation axis, and *B_9_* + *B_10_* are the trunnion axis wobble.

**Figure 7.**
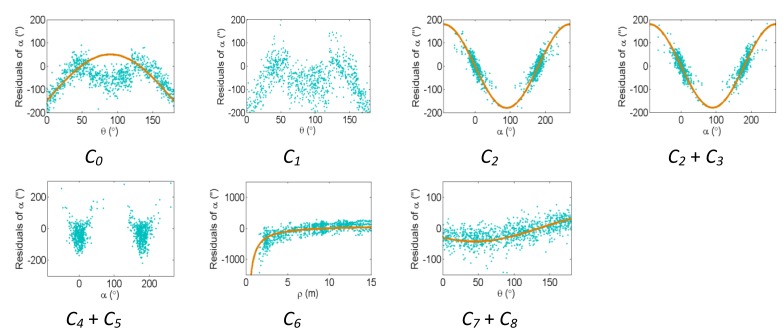
Residual plots of systematic errors in the vertical angle observations (*α*) from point-based self-calibration for panoramic-type scanners: *C_0_* is the vertical circle index error, *C_1_* is the scale factor error, *C_2_* + *C_3_* are the vertical circle eccentricity, *C_4_* + *C_5_* are the non-orthogonality of vertical encoder and trunnion axis, *C_6_* is the vertical eccentricity of collimation axis, and *C_7_* + *C_8_* is the vertical axis wobble.

Figures 2, 3 and 4 show the effect of the systematic error terms in distance, horizontal direction, and vertical angle observations respectively (as presented in Equations 4–6) for hybrid scanners using point-based self-calibration. Similar plots showing the propagation of systematic errors into the observation residuals for panoramic type scanners are shown in Figures 5, 6 and 7. Whenever a pattern in the residuals is visually identifiable and follows the mathematical model, a curve has been superimposed. Based on graphical analysis of these observation residual plots it is more difficult to identify the systematic error terms for scanners that can only observe in one face (*i.e.*, hybrid scanners); in Figure 4 only the trend of C_7_ and C_8_ can be visually identified while in Figure 7 trends for all APs can be identified. The majority of errors in the vertical direction cannot be identified by visual inspection of the plots in the case of hybrid scanners. Typically, error modelling of hybrid type scanners is more based on a trial-and-error approach with statistical tests and RMSE of residuals indicating parameter significance. The appearance of *C_0_* in the residuals for panoramic-type scanners is slightly different from the plot reported in [11]. This difference exists because its exact pattern is dependent on the network geometry. It is worth mentioning that the *C_3_* term was included in the error model, despite the fact that it was omitted in [18] due to its high correlation with other parameters. It will be shown later that this correlation issue can be mitigated with additional observations and/or tilting the scanner.

**Figure 8.**
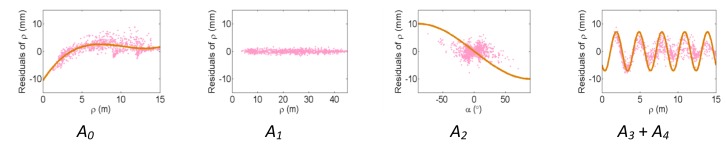
Residual plots of systematic errors in the range observations (*ρ*) from plane-based self-calibration for hybrid-type scanners: *A_0_* is the rangefinder offset, *A_1_* is the range scale factor error, *A_2_* is the laser axis vertical offset, and *A_3_* + *A_4_* are the cyclic errors.

**Figure 9.**
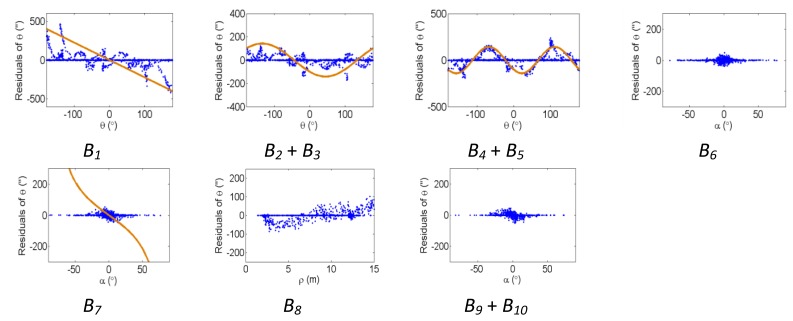
Residual plots of systematic errors in the horizontal angle observations (*θ*) from plane-based self-calibration for hybrid-type scanners: *B_1_* is the horizontal direction scale factor error, *B_2_* + *B_3_* are the horizontal circle eccentricity, *B_4_* + *B_5_* are the non-orthogonality of horizontal encoder and vertical axis, *B_6_* is the horizontal collimation axis error, *B_7_* is the trunnion axis error, *B_8_* is the horizontal eccentricity of collimation axis, and *B_9_* + *B_10_* are the trunnion axis wobble.

**Figure 10.**
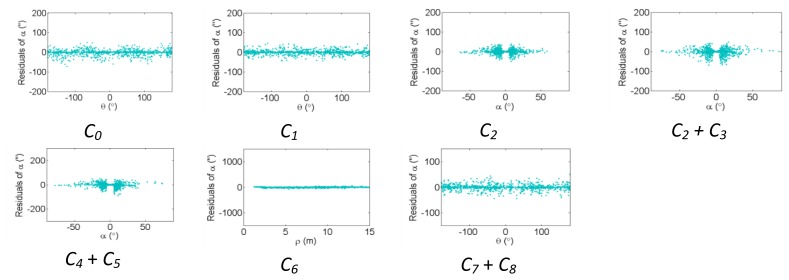
Residual plots of systematic errors in the vertical angle observations (*α*) from plane-based self-calibration for hybrid-type scanners: *C_0_* is the vertical circle index error, *C_1_* is the scale factor error, *C_2_* + *C_3_* are the vertical circle eccentricity, *C_4_* + *C_5_* are the non-orthogonality of vertical encoder and trunnion axis, *C_6_* is the vertical eccentricity of collimation axis, and *C_7_* + *C_8_* is the vertical axis wobble.

**Figure 11.**
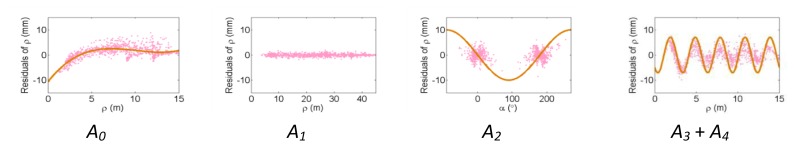
Residual plots of systematic errors in the range observations (*ρ*) from plane-based self-calibration for panoramic-type scanners: *A_0_* is the rangefinder offset, *A_1_* is the range scale factor error, *A_2_* is the laser axis vertical offset, and *A_3_* + *A_4_* are the cyclic errors.

**Figure 12.**
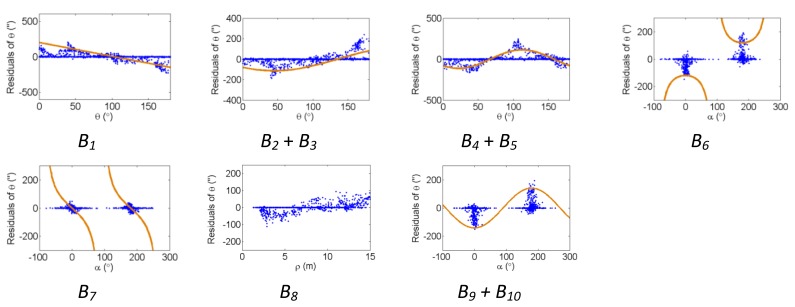
Residual plots of systematic errors in the horizontal angle observations (*θ*) from plane-based self-calibration for panoramic-type scanners: *B_1_* is the horizontal direction scale factor error, *B_2_* + *B_3_* are the horizontal circle eccentricity, *B_4_* + *B_5_* are the non-orthogonality of horizontal encoder and vertical axis, *B_6_* is the horizontal collimation axis error, *B_7_* is the trunnion axis error, *B_8_* is the horizontal eccentricity of collimation axis, and *B_9_* + *B_10_* are the trunnion axis wobble.

**Figure 13.**
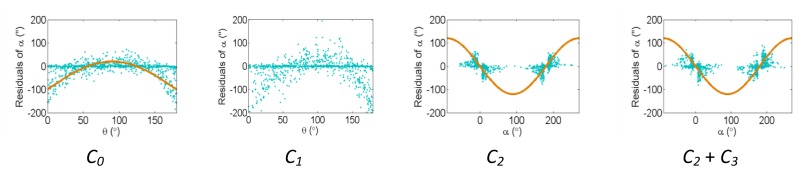

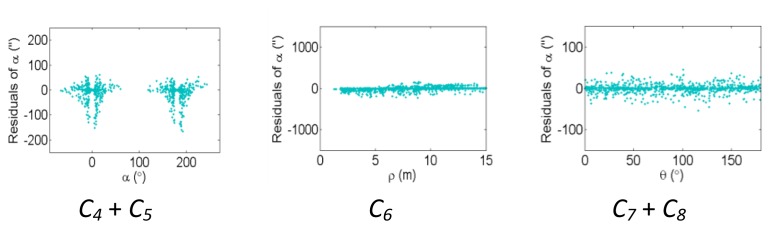
Residual plots of systematic errors in the vertical angle observations (*α*) from plane-based self-calibration for panoramic-type scanners: *C_0_* is the vertical circle index error, *C_1_* is the scale factor error, *C_2_* + *C_3_* are the vertical circle eccentricity, *C_4_* + *C_5_* are the non-orthogonality of vertical encoder and trunnion axis, *C_6_* is the vertical eccentricity of collimation axis, and *C_7_* + *C_8_* is the vertical axis wobble.

The residual plots from the same point clouds processed using the plane-based self-calibration are shown in Figures 8, 9 and 10 for hybrid-type scanners, and Figures 11, 12 and 13 for panoramic-type scanners.

Points that are observed with a small incidence angle give rise to many zero-valued residuals in the angular measurements, which is an undesirable trait of plane-based self-calibration. These observations bias the statistics computed from the residuals and do not provide useful information for systematic error identification/modelling. Geometrically speaking, planar features can only constrain points in 1D, while point features are well-controlled in 3D. Regardless of the number of points in the point cloud, the average redundancy never exceeds 33% due to the nature of planes. The rangefinder offset is highly dependent on the incidence angle, and can only be measured from points observed with a small incidence angle [26]. When studying the internal reliability of each observation, it was clear that for the range observations the individual redundancy number decreases with increasing incidence angle and distance to target, while the opposite holds true for the angular observations. The *B_6_* term is completely hidden for hybrid-type scanners under both calibration routines. In this network configuration, some APs (e.g., *B_7_* and *B_9_* + *B_10_* for hybrid scanners, and *B_6_* and *B_9_* + *B_10_* for panoramic scanners) gave rise to similar trends in the residual plots and are difficult to distinguish.

The corresponding residual plots between the point-based and plane-based self-calibrations for both scanner architectures are similar, suggesting that they can yield comparable results. However, there are some downfalls for the plane-based calibration — for example, the vertical eccentricity of collimation axis (*C_6_*) and wobbling (*C_7_* and *C_8_*) do not propagate into residual plots under the tested network geometry. As mentioned, visual identification of trends in the observation residuals can be an indication that the systematic error is decoupled from other observations and thus can be modelled in the observation space with greater confidence. Systematic error terms such as *B_8_* (horizontal eccentricity of collimation axis) in Figures 3 and 6 appear differently than the similar *C_6_* term (vertical eccentricity of collimation axis). Despite the similarities between the error models, yet different behavior in the observation residuals, both terms were recovered accurately. In general, systematic errors that were identifiable in the observation residual plots were accurately recovered using the calibration methods presented, including *C_0_* in hybrid-type scanners. Most systematic error terms tested to this point can be identified in the residual plots for panoramic type scanners, regardless of the primitives used for calibration. However, the same cannot be said for hybrid type scanners. In particular, there is poor recovery of vertical angular errors (Figures 4 and 10). The following section presents a feasible solution to these limitations of point-based calibration and in particular addresses the issue of solving for *B_6_* in hybrid-type scanners.

### 4.1. Tilted Scans

**Figure 14.**
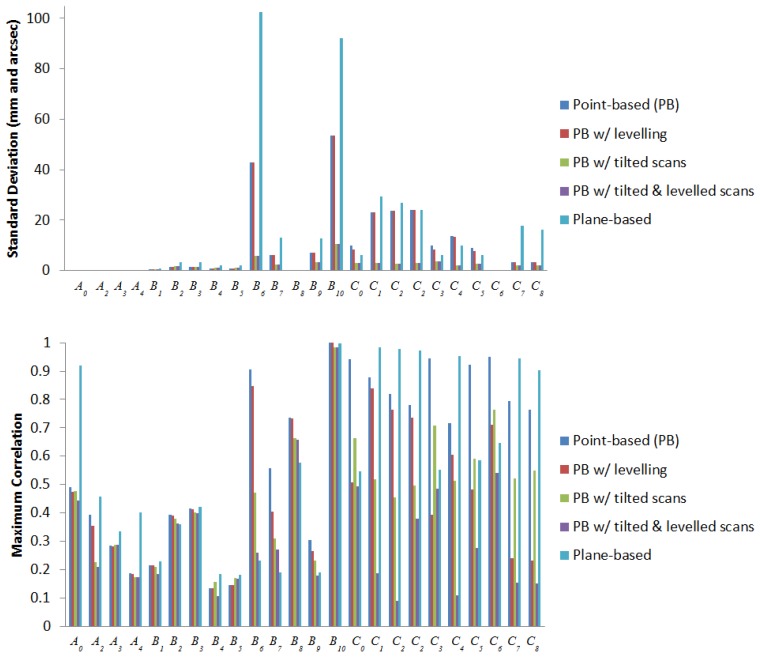

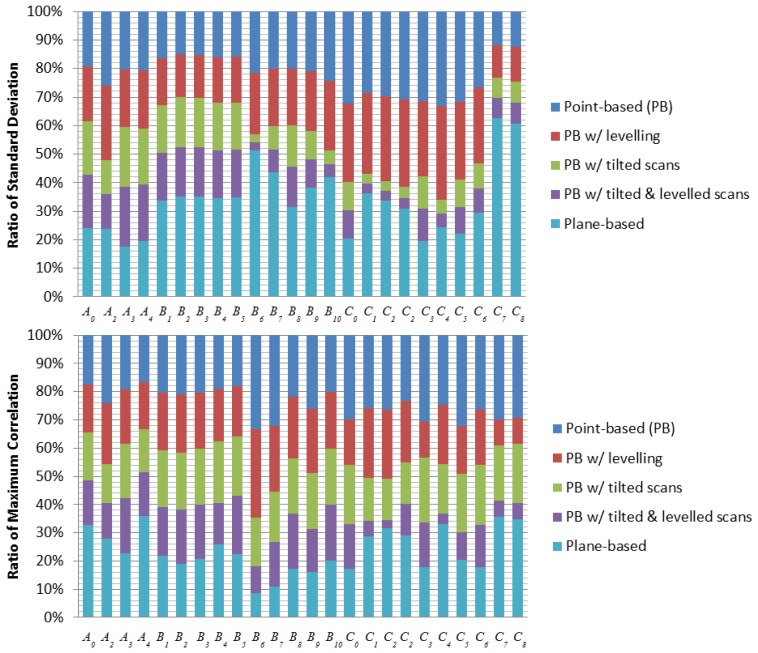
Standard deviation and maximum correlation of the recovered APs for hybrid-type scanners in simulation.

**Figure 15.**
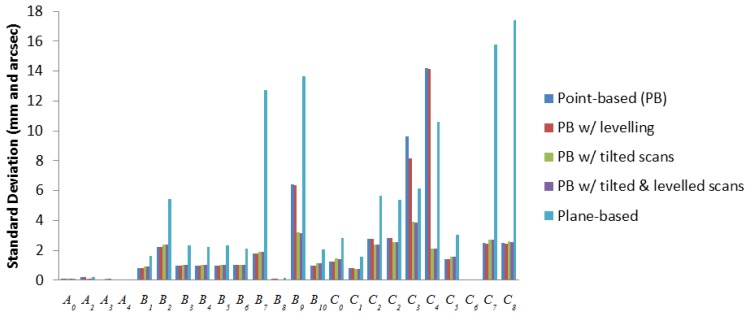

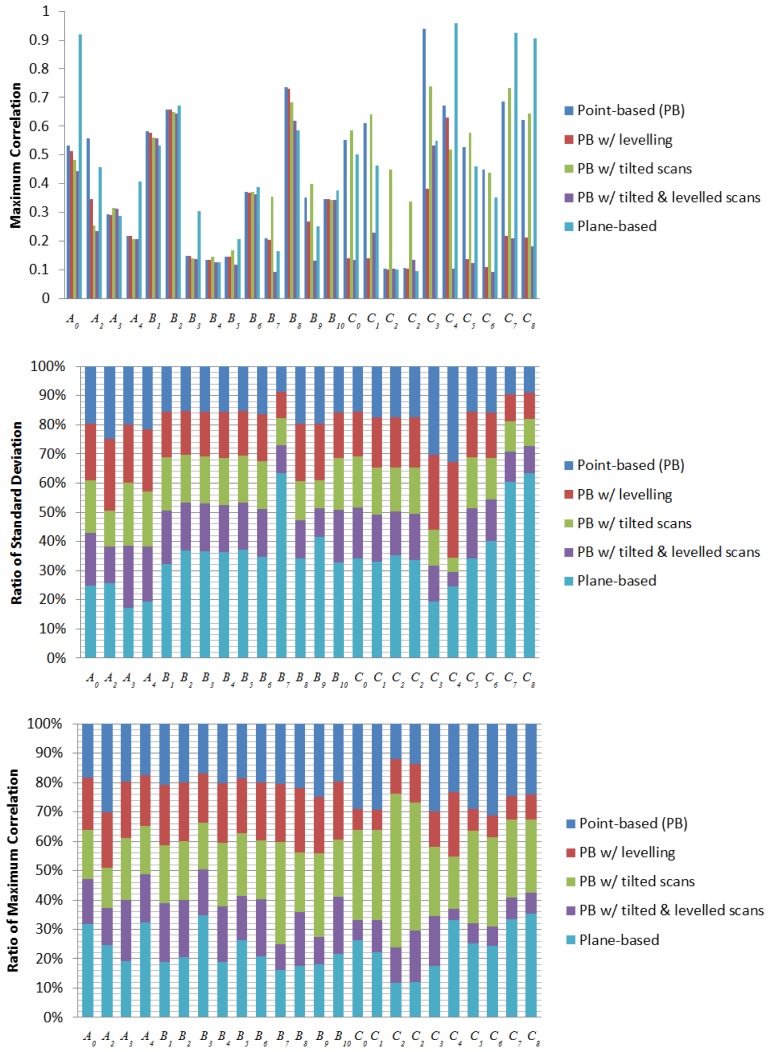
Standard deviation and maximum correlation of the recovered APs for panoramic-type scanners in simulation

The main advantage in self-calibration of panoramic scanners over hybrid scanners is observation of data in two layers, where vertical angle observations in front of and behind the scanner are in different quadrants. This implicitly decouples several systematic errors from other parameters because they have the same magnitude but opposite sign on opposing scanner faces. For hybrid-type scanners, a similar decoupling effect can be achieved by tilting the scanner with a roll angle of 90° as is done in photogrammetric self-calibrations [23]. To be consistent with the data captured in the real experiment, scans with a roll angle of −45° and +45° were simulated instead. Everything else in the simulation remains the same as before, with the replacement of two tilted scans at one of the scan stations. As shown in Figures 14 and 15, the desired correlation reduction is achieved, even for the horizontal collimation axis error in hybrid scanners.

The standard deviations of the recovered systematic errors (given in millimetres and arc seconds) are reduced. It is worth mentioning that in [19] it was reported that beyond a fairly low threshold, additional scans or targets give only minimal improvement to the TLS self-calibration. Hence, the improvements being observed here are mainly a result of the geometric strength gained from tilting the scanner. Combining tilted scans with levelling constraints brings forth additional improvement to the correlations on all the vertical angular errors for hybrid scanners, with the exception of *C_3_*. The standard deviation and correlation of *B_6_* and *B_10_* for hybrid scanners benefitted the most from tilting the scanner. The standard deviations dropped by 86% and 80% respectively; however, the maximum correlation for *B_10_* remained high. For panoramic scanners, their APs had better standard deviation and correlation than hybrid scans, and the only significant improvement to the standard deviations from tilting the scanner are found in *B_9_*, *C_3_* and *C_4_* (50%, 60% and 85%, respectively). Most APs recovered in this simulation using planar features suffer from lower precision and higher correlation than the point-based calibration. However, it can be seen that most APs recoverable by point-based calibration are also recoverable by plane-based calibration.

## 5. Real Datasets: Results and Discussion

Point clouds captured by scanners with hybrid architecture (*i.e.*, a Riegl VZ-400 and a Leica HDS6100 operating in hybrid mode) and panoramic architecture (*i.e.*, a Z+F Imager 5003 and a HDS6100 operating in panoramic mode) are used for testing the proposed methodology. We first present the influence of tilted scans on the calibration of the VZ-400 and the Imager 5003. This will be followed by a comparison of the point-based calibration to plane-based calibration with real data, showing that they can yield the same results. For a fair comparison, the same point clouds were used in both point-based and plane-based self-calibrations with the only difference being the extracted primitives (*i.e.*, signalised targets or planes).

### 5.1. Tilted Scans

**Figure 16.**
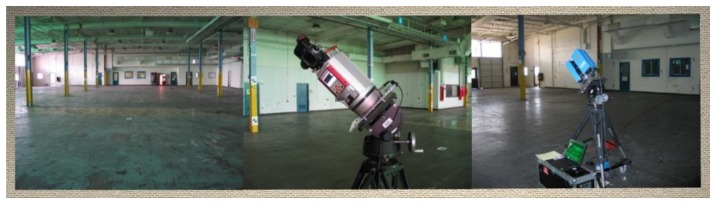
TLS user self-calibration field setup with a Riegl VZ-400 and Z+F Imager 5003 at the University of Houston.

The Riegl VZ-400 and the Z+F Imager 5003 were calibrated in a 30 m by 33 m by 5 m room located at the University of Houston (Figure 16). A specialized tripod was used for acquiring scans on a -45° and +45° incline. Checkerboard-type paper targets were affixed to the ceiling, floor, wall, and the vertical support I-beams. The statistically significant systematic errors found in the calibration of the Riegl VZ-400 and the Z+F Imager 5003 with and without the tilted scans are shown in Tables 1 and 2, respectively.

These APs were selected based on a combination of graphical analysis of the observation residual plots and statistical analysis. The calibration setup had targets covering distances from 3 m to 30 m, which is a desirable trait for range calibrations. However, due to the poor distribution of targets near the extrema of the vertical FOV and the scanner being only approximately levelled, the correlations between the APs and other parameters are rather high. The largest standard deviation in the above tables is for the horizontal collimation axis error (*B_6_*) in the VZ-400. Although this error is statistically significant based on the t-test, the magnitude of the error appears to be unreasonably high. Even though the reduced horizontal collimation axis error model was used, it is expected to have no effect on the standard deviation as previously shown [11]. With the reduced model, the perfect correlation with the tertiary rotation angle has been removed, but it is still highly correlated with other parameters (e.g., up to 0.98 with object space target coordinates). By including two tilted scans, the maximum correlation of all the APs were reduced. Correlations with the *B_6_* and *B_7_* terms received the most benefit as they were reduced by 37% and 68%, respectively. The magnitude of the recovered horizontal angular errors was reduced drastically to a more reasonable level and the standard deviations for *B_6_* and *B_7_* were improved by 97% and 96%, respectively. This is lower than any standard deviation for *B_6_* found in literature for hybrid-type scanners.

The standard deviation of all the calibrated angular parameters is lower for the panoramic scanners than for the hybrid scanners, as expected. In this network configuration, where the vertical distribution of targets is not ideal and dual-axis compensation was not used for the scanners, tilted scans significantly improved the quality of the calibration. The improvements are even more pronounced than in the simulated results, where the targets were better spread out within the scanner's FOV.

### 5.2. Comparison of Point-Based and Plane-Based TLS Self-Calibration

**Figure 17.**
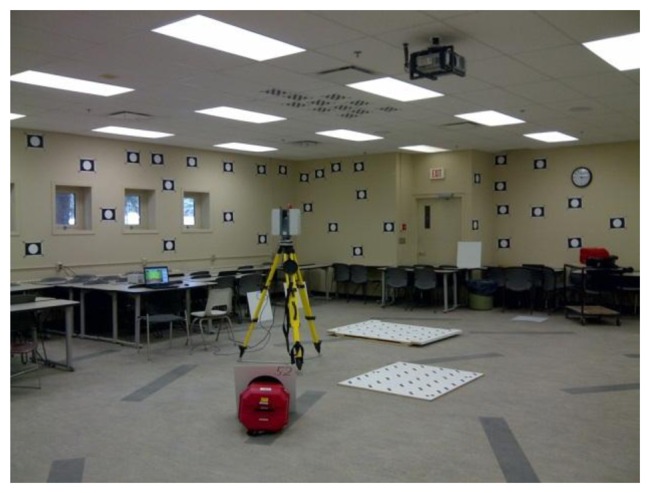
TLS user self-calibration at the University of Calgary.

Point-based and plane-based calibration results for the Leica HDS6100 in hybrid mode and in panoramic mode are presented in Tables 3 and 4, respectively. No tilted scans were included, but a large number of targets and planes with various orientations were used. Data were captured in a 14 m by 11 m by 3 m room at the University of Calgary (Figure 17). Special care was taken to ensure a large number of observations were made above the scanner and as close to the tripod legs as possible.

Evaluating the success of the point-based self-calibration is quite intuitive. For instance a check point analysis can be performed, or the RMSE of the residuals and/or the standard deviation of the observations estimated by VCE can be compared before and after error modeling [18]. On the other hand, evaluating the plane-based self-calibration is less intuitive. Planes are difficult to survey with accuracy at least an order of magnitude better than the TLS results, especially since some TLS instruments can measure ranges with sub-millimetre accuracy at close-range. As mentioned in Section 2, statistics computed from the residuals are biased because of the large amount of zero residuals. At close-range the improvements to the misclosure vector obtained from calibration are fairly small and are not an appropriate metric for evaluation of the error modeling. Therefore, in this paper the plane-based self-calibration is deemed effective when the recovered APs are comparable to the point-based self-calibration results. Since naively comparing the individual APs separately has been shown to be a flawed approach [36], comparing the APs as a group through simulation is used instead [37]. In all APs comparisons below, 1,000 simulations in a rectangular room having the same dimensions and same observational noise as the real data were tested. A total of 100 targets at different distances within the measurement volume were tested with 10 points chosen to be used as control.

In hybrid mode, the HDS6100 can observe up to the zenith, and by densifying the targets to cover the extrema of vertical angles, the horizontal collimation axis error can be recovered with a standard deviation of a few arc seconds. This is even lower than the value reported in Table 1, with one of the drawbacks being that the maximum correlation is higher than when tilted scans were added. It appears that if the hybrid scanner has a wide enough vertical field of view, then the four fundamental systematic errors can be recovered with high precision without any special constraints other than organizing the targets to have a good coverage at the maximum and minimum vertical angles. This finding has been confirmed through simulation. Using the same point clouds and calibrating the scanner using planar primitives, a slightly different set of APs were recovered. A difference in *A_0_* should not come as a surprise since the rangefinder offset is known to be a function of the scanner, target fitting algorithm, and properties of the target. Based on the APs compatibility test described earlier, the AP sets recovered using either primitive were found to be comparable for the HDS6100 (Tables 3 and 4). The results are similar regardless of whether the scanner was levelled using the built-in dual axis compensator and the levelling constraints were applied or not. In the plane-based self-calibration of the HDS6100, it is worth mentioning that planes with various orientations were used, and this led to parameter decoupling, as indicated by the low correlation of the APs. If the vertical FOV is restricted due to the scanner design, or targets could not be placed at the vertical extrema of the scanner, then tilted scans appears to be a viable alternative. Table 5 shows the plane-based calibration results of the Imager 5003 using the same data as shown in Table 2. With tilted scans the maximum correlations in the plane-based calibration are reduced for *B_6_* and *B_7_*, at the expense of inflating the correlation for *A_0_* and *C_0_* when compared to only using levelled scans; however the precision of all the recovered systematic errors is improved. More importantly, when compared to the point-based calibration results, only the group of APs estimated with tilted scans is comparable.

Not only does this show that the two methods can give similar results, some benefits of plane-based self-calibration are also realized. In general, when accounting for only the four fundamental systematic errors, the plane-based self-calibration can yield the same level or better standard deviations and correlations. This, along with the expedited workflow from data capture (*i.e.*, no need to prepare a room full of signalized targets) to processing (*i.e.*, automatic plane identification and matching), makes it the more desirable calibration method of the two.

## 6. Conclusions

The self-calibration of TLS instruments using point and planar primitives has been studied. Through simulated and real data, it has been demonstrated that having tilted scans in the network can improve the point-based self-calibration results, especially for hybrid scanners. For the first time, the horizontal collimation axis error in hybrid scanners was solved with reasonable correlation and standard deviation. If the hybrid scanner has a large field of view, the collimation axis error can even be recovered with low standard deviation by placing targets near zenith and the tripod legs. Tilted scans were also applied to the calibration of a panoramic scanner, and improvements in the quality of the recovered APs in both point-based and plane-based calibrations were observed.

Although plane-based calibration has shown potential to be a more efficient candidate for modelling systematic errors in laser scanners, some noticeable drawbacks such as poorer recoverability of vertical angular errors have been identified. In simulation, when given identical data, plane-based calibration resulted in higher standard deviations and correlations than point-based calibration. However, it was shown using real datasets that if some diversity exists in the plane orientations, then plane-based calibration can deliver a similar set of APs as the better studied point-based calibration. It can also achieve lower standard deviations and almost complete parameter de-coupling for the four fundamental systematic errors.

The results of this work can help improve the usability of TLS instruments by increasing the geometric accuracy of the data and reducing the need to send the scanner to the manufacturer for tuning. In applications that require 3D reconstruction and/or geometric modelling (e.g., infrastructure documentation) the fit between the observations and the model can be improved. In addition, the overall processing time may be decreased due to reduced necessity of point cloud filtering/smoothing [6]. For deformation monitoring it can even lead to higher detection sensitivity. Improved point cloud accuracy can also reduce errors during point cloud classification. Future research will focus on the attainable quality of the plane-based self-calibration routine under various network geometries and study ways of improving and independently validating the results from this method.

## Figures and Tables

**Table 1.** Riegl VZ-400 point-based self-calibration.

	**Without Tilted Scans**			**With Tilted Scans**		
**# of Targets**	61			61		
**# of Scans**	4 (levelled)			4 (levelled) + 2 (tilted)		
**# of Obs.**	456			639		
**# of Unk.**	211			422		
***ρ_min_***, ***ρ_max_*[m]**	3.3, 30.6			3.3, 30.6		
***α_min_***, ***α_max_*[°]**	−24, 35			−40, 47		
	**Value**	**σ**	**Max Abs. Correlation (w/parameter)**	**Value**	**σ**	**Max Abs. Correlation (w/parameter)**

***A_0_*[mm]**	2.8	0.7	0.92 (*Y*)	2.6	0.4	0.82 (*X*)
***C0* [“]**	-16.1	20.6	1.00 (*Z_o_*)	9.6	3.9	0.88 (*Z_o_*)
***B_6_* [“]**	3318.0	927.3	0.98 (*X*)	109.5	24.1	0.62 (*κ*)
***B7* [“]**	472.0	131.4	0.92 (*Y*)	23.1	5.3	0.29 (*Y_o_*)

**Table 2.** Z+F Imager 5003 point-based self-calibration.

	**Without Tilted Scans**			**With Tilted Scans**		
**# of Targets**	65			67		
**# of Scans**	4 (levelled)			4 (levelled) + 2 (tilted)		
**# of Obs.**	390			480		
**# of Unk.**	223			241		
***ρ_min_, ρ_max_* [m]**	4.0, 30.6			3.3, 30.6		
***α_min_, α_max_* [°]**	−16.5, 199			−49, 235		
	**Value**	**σ**	**Max Abs. Correlation (w/parameter)**	**Value**	**σ**	**Max Abs. Correlation (w/parameter)**

***A_0_* [mm]**	3.6	1.1	0.96 (*Y*)	−0.6	0.5	0.83 (*Z*)
***C_0_* [“]**	40.5	8.3	0.90 (*ω*)	36.9	5.3	0.77 (*ø*)
***B_6_* [“]**	3.5	3.6	0.49 (*X*)	−6.0	2.5	0.30 (*Y_o_*)
***B_7_* [“]**	79.7	35.4	0.67 (*Y*)	−19.7	9.5	0.47 (*κ*)

**Table 3.** Self-calibration of the Leica HDS6100 in hybrid mode.

	**Point-Based Self-Calibration**			**Plane-Based Self-Calibration**		
**# of Targets/Planes**	285			118		
**# of Scans**	6			6		
**# of Obs.**	3405			98982		
**# of Unk.**	895			512		
***ρ_min_*, *ρ_max_* [m]**	1.1, 15.3			1.2, 15.2		
***α_min_*, *α_max_* [°]**	-60, 88			-64, 58		
	**Value**	**σ**	**Max Abs. Correlation (w/parameter)**	**Value**	**σ**	**Max Abs. Correlation (w/parameter)**

***A_0_* [mm]**	−0.6	0.05	0.62 (*X_o_*)	−0.5	0.02	0.17 (*B_6_*)
***C_0_* [“]**	−87.3	3.2	0.94 (*Z_o_*)	−77.5	1.5	0.11 (*ø*)
***B_6_* [“]**	−24.2	5.8	0.87 (*Z_o_*)	−27.6	12.8	0.48 (*B_7_*)
***B_7_* [“]**	20.3	4.6	0.58 (*X*)	40.0	2.4	0.48 (*B_6_*)

**Table 4.** Self-calibration of the Leica HDS6100 in panoramic mode.

**Dataset 1**						
	**Point-Based Self-Calibration**			**Plane-Based Self-Calibration**		
**# of Targets/Planes**	285			118		
**# of Scans**	6			6		
**# of Obs.**	3039			98877		
**# of Unk.**	895			512		
***ρ_min_*, *ρ_max_* [m]**	1.1, 15.4			1.1, 15.2		
***α_min_*, *α_max_* [°]**	−59, 240			−64, 244		
	**Value**	**σ**	**Max Abs. Correlation (w/parameter)**	**Value**	**σ**	**Max Abs. Correlation (w/parameter)**

***A_0_* [mm]**	−0.8	0.05	0.59 (*Y_o_*)	−0.5	0.02	0.12 (*B_6_*)
***C_0_* [“]**	10.7	1.3	0.55 (*ω*)	3.8	0.5	0.09 (*B_6_*)
***B_6_* [“]**	−2.5	0.7	0.29 (*Y_o_*)	1.8	0.2	0.12 (*A_0_*)
***B_7_* [“]**	−48.6	1.9	0.31 (*Y*)	−57.0	1.8	0.06 (*B_6_*)
**Dataset 2 (Levelled via dual-axis compensator)**						
	**Point-Based Self-Calibration**			**Plane-Based Self-Calibration**		

**# of Targets/Planes**	384			223		
**# of Scans**	5			5		
**# of Obs.**	2878			126567		
**# of Unk.**	1186			926		
***ρ_min_*, *ρ_max_* [m]**	1.1, 15.5			1.1, 14.0		
***α_min_*, *α_max_* [°]**	−62, 239			−64, 240		
	**Value**	**σ**	**Max Abs. Correlation (w/parameter)**	**Value**	**σ**	**Max Abs. Correlation (w/parameter)**

***A_0_* [mm]**	−1.7	0.1	0.72 (*Y_o_*)	−0.4	0.1	0.08 (*B_7_*)
***C_0_* [“]**	10.7	2.0	0.16 (*Ø*)	8.1	1.5	0.08 (*B_7_*)
***B_6_* [“]**	−19.4	1.4	0.23 (*Y_o_*)	−4.5	0.5	0.09 (*B_7_*)
***B_7_* [“]**	−84.8	2.1	0.22 (*Y*)	−69.4	4.0	0.09 (*B_6_*)

**Table 5.** Z+F Imager 5003 plane-based self-calibration.

	**Without Tilted Scans**			**With Tilted Scans**		
**# of Targets/Planes**	109			114		
**# of Scans**	4			6		
**# of Obs.**	50781			63507		
**# of Unk.**	464			496		
***ρ_min_*, *ρ_max_* [m]**	2.2, 24.1			2.2, 33.8		
***α_min_*, *α_max_* [°]**	−57, 236			−65, 244		
	**Value**	**σ**	**Max Abs. Correlation (w/parameter)**	**Value**	**σ**	**Max Abs. Correlation (w/parameter)**

***A_0_* [mm]**	0.8	0.1	0.06 (*B_6_*)	−1.0	0.1	0.15 (*B_6_*)
***C_0_* [“]**	18.6	1.8	0.06 (*ω*)	−0.3	1.4	0.33 (*B_7_*)
***B_6_* [“]**	1.8	0.8	0.49 (*B_7_*)	−3.3	0.6	0.11 (*B_7_*)
***B_7_* [“]**	22.9	7.5	0.49 (*B_6_*)	−9.1	2.0	0.33 (*C_0_*)
